# Different general strategies for deprescribing in real clinical settings: From lists to collaborative care

**DOI:** 10.1192/j.eurpsy.2021.182

**Published:** 2021-08-13

**Authors:** M. Stuhec

**Affiliations:** 1 Clinical Pharmacy & Pharmacology, University of Maribor, Medical Faculty, Maribor, Slovenia; 2 Clinical Pharmacy, University of Ljubljana, Faculty of Pharmacy & Ormoz Psychiatric Hospital, Ormoz, Slovenia

**Keywords:** Psychopharmacology, Collaborative care, Deprescribing, Real Clinical Setting

## Abstract

Most elderly patients with mental disorders are treated with polypharmacy (e.g., five or more medications), and they are receiving medications that are potentially inappropriate for elderly patients (e.g., PIMs). These aspects are often excluded in the clinical guidelines, meta-analyses, and randomized controlled trials but are very important for prudent prescribing in daily practice. The most robust approach to reducing irrational polypharmacy, PIMs, and other medications-related problems in this population is a careful deprescribing process. It is the process of tapering, withdrawing, discontinuing, or stopping medications. There are some tools available to help in the deprescribing process in clinical practice, including different medication lists (e.g., Beers criteria, STOPP/START, and guidelines) and collaborative care, including clinical pharmacist or pharmacologist. Medication lists have been used in clinical trials and guidelines, where Beers criteria are used predominantly in the U.S. and Priscus list in Europe. A collaborative care approach, including a clinical pharmacist, has been established only in some countries (e.g., USA, UK & Slovenia). The results are positive with a decrease of PIMs, polypharmacy, and an increase in the patients’ quality of life. The participants will learn the general deprescribing processes supported by the evidence-based data and real clinical pharmacological tools useful for daily practice.

**Disclosure:**

No significant relationships.

